# Impact of the COVID-19 pandemic’s first wave on the care and treatment situation of intravitreal injections in a German metropolitan region

**DOI:** 10.1007/s00417-021-05521-5

**Published:** 2022-01-10

**Authors:** Birthe Stemplewitz, Joel Luethy, Mau-Thek Eddy, Martin Spitzer, Ulrike Brocks, Julie Kieckhoefel, Christa Schneemann, Ulrich Schaudig, Marc Schargus

**Affiliations:** 1 Department of Ophthalmology, Asklepios Hospital Barmbek, Hamburg, Germany; 2 Department of Ophthalmology, Asklepios Hospital Nord-Heidberg, Hamburg, Germany; 3 Department of Ophthalmology, Asklepios Hospital Altona, Hamburg, Germany; 4 Department of Ophthalmology, University Medical Center Hamburg-Eppendorf, Hamburg, Germany

**Keywords:** Intravitreal injections, COVID-19, Pandemic, Adherence

## Abstract

**Purpose:**

This study aims to evaluate the impact of the first coronavirus 2019 (COVID-19) wave in 2020 on patients scheduled for intravitreal injections (IVI) in a German metropolitan region.

**Methods:**

We performed a multicentre prospective survey and retrospective analysis of the records of patients treated with intravitreal injections during the 20-week period from March to July 2020 in all four hospital eye departments in the city of Hamburg using a questionnaire (on treatment adherence, SarsCoV2-related personal, familial and social data) and treatment data.

**Results:**

A total of 1038 patients (2472 IVI, 1231 eyes) and 818 questionnaires were evaluated. Longer duration of therapy, lower visual acuity (VA) of the treated and higher VA of the fellow untreated eye was were associated with a higher probability of visit cancellation. Every additional year of life posed a 2.6% lower risk of noncompliance. A COVID-19 infection in the family environment displayed a 5.5-fold chance of visit cancellation. Patients treated for neovascular age-related macular degeneration (nAMD) had a 36% reduced risk of visit cancellation compared to patients with diabetic macular oedema (DME).

**Conclusion:**

A long preceding treatment period, low VA of the treated eye, high VA of the untreated eye, COVID-19 in the family and DME were identified as risk factors for IVI visit cancellations during the COVID-19 pandemic. Compliance to treatment might be improved in the future by taking these risk factors into account when scheduling patients for IVI during the exceptional circumstances of a pandemic.

**Supplementary Information:**

The online version contains supplementary material available at 10.1007/s00417-021-05521-5.


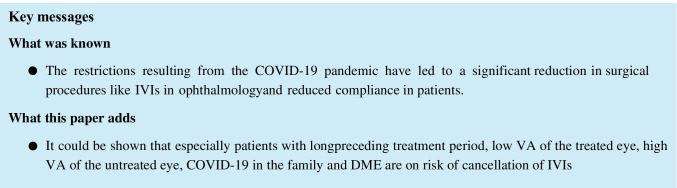


## Introduction

The COVID-19 pandemic, caused by the SARS-CoV-2 virus, has led to limited healthcare in every medical branch across the world due to limited and held back capacities, government restrictions and increased patient noncompliance.

The major impact of the pandemic on the number of hospital visits has been reported worldwide both for life-threatening events such as stroke [1–5]. SARS-CoV-2 caused the biggest pandemic since “the Spanish flu” by influenza virus H1N1 in 1918, and at its beginning, it was difficult to predict in detail the extent of reduced medical care, even in a modern medical healthcare setting [6].

From an ophthalmologist’s point of view, the effect of COVID-19 on emergency and elective medical treatment needs to be analysed. This could be helpful to predict problems and improve patients’ compliance in the future.

In Germany, intravitreal injections (IVI) are by far the most frequently performed medical procedure (approximately 1.2 million injections per year) and account for up to 54% of all intraocular surgeries [7, 8].

Therefore, the extent of the consequences of the COVID 19 pandemic on the administration of intravitreal injections needs evaluation.

The most common indications of IVI are neovascular age-related macular degeneration (nAMD), diabetic macular oedema (DME), macular oedema caused by retinal vein occlusion (RVO) and myopic choroidal neovascularization or other causes. The course of these diseases is monitored by best-corrected visual acuity (BCVA), central retinal thickness and retinal morphology (CRT) using optical coherence tomography (OCT) and fluorescence angiography [9–12].

All these diseases, if left untreated, can lead to profound visual impairment. Large studies early in the pandemic showed that older patients and patients with comorbidities are at higher risk for severe COVID-19. This matches the predominant age group for treatment with IVI (Wu et al. 2020).

Anti-vascular endothelial growth factor (Anti-VEGF) intravitreal injections are usually scheduled following a pro re nata (PRN) or treat-and-extend (T&E) protocol with intervals of 4–12 weeks. National and international ophthalmic societies and expert panels recommended to continue treatment plans despite COVID-19-related restrictions [13, 14], concern of substantial pandemic-related cancellations was reported early on (Fung et al. 2020), and later studies confirmed the short-term risk for visual impairment due to interrupted treatment [15–17].

Wasser et al. reported a 36% drop of the number of IVI when comparing the 4-week period between March and April 2020 to 2019. Their total number of IVI decreased 58% from February 20th to April 1st, 2020, while governmental restrictions increased [18]. Borelli et al. revisited the charts of their outpatient retina clinic during the first 8 weeks (March to May 2020) of the pandemic in Italy and found a 53.6% reduction of intravitreal injections compared to the same period in 2019 [19], and Carnevali et al. even reported a 91% decrease during the COVID-19 pandemic [20].

To the best of our knowledge, there is no data on the effect of COVID-19 on intravitreal injections correlating to individual patients and identifying risk factors for noncompliance. Those risk factors might include age, sex, IVI treatment diagnosis, bilateral IVI, IVI treatment duration and preoperative visual acuity of the treated or of the untreated eye.

## Methods

### Ethics and approval

The study followed the demands of the Declaration of Helsinki and was approved by the local ethics committee of the Medical Association of Hamburg (Ärztekammer Hamburg).

### Study period

To gather sufficient and representative data of intravitreal injections, we chose a study period of 20 weeks, starting on March 15th, 2020.

The time period coincided with the official declaration of a pandemic by the World Health Organization (WHO) on March 11th, 2020 [6], and with the ordered restrictions by the German government starting in March 2020.

Consequently, the retrospective study period was March 15th to July 31st, 2020.

Treatment with intravitreal injections were continued despite restrictions of the health authorities throughout the pandemic.

In all hospitals, general measures for the safety of patients and personal were implemented throughout the study period. Patient-to-patient distances were increased, protective shields on slit lamps were mounted, patient time during the procedure was reduced, and the personnel wore face masks at any time.

### Patients

We consecutively recruited all patients from all four hospitals with a department of ophthalmology within the city state of Hamburg: Asklepios Klinik Nord-Heidberg (AKN), Asklepios Klinik Barmbek (AKB), Asklepios Klinik Altona (AKA) and the Department of Ophthalmology of the University Medical Center Hamburg-Eppendorf (UKE).

Inclusion criteria included written informed consent, at least one intravitreal injection in the study period between March 15th and July 31st, 2020, and an indication for intravitreal injections according to current German guidelines. The examination consisted of an ophthalmic examination including best-corrected visual acuity, fundoscopy, optical coherence tomography and fluorescein angiography (FA), if necessary. Exclusion criteria were inability to consent, missing consent and withdrawal of consent. Patient data like treatment diagnosis, number of previously received intravitreal injections and treatment period per eye, as well as every IVI with date and substance within our study period, were obtained from patient records of each hospital. In addition, the appointment cancellation rate by patients during this period was analysed from the clinics’ appointment diaries and compared with the cancellation rates from the same period in 2019.

In addition, patients were asked to complete a custom-made questionnaire to gain further information about COIVD-19-related healthcare issues. Questionnaires were filled out between September 8th and December 21st, 2020, in collaboration with a research assistant either in person during a treatment appointment or via telephone. Patients who could not be reached in person or via phone were contacted by mail, asked to complete the questionnaires and return it with a labeled envelope.

### Questionnaire

The German questionnaire (translated in Supplement 1) consisted of 28 questions on two pages and was divided in three sections:

1. Infectious status regarding COVID-19
2. Ophthalmological treatment during COVID-19
3. Home and medical care situation

Patients were asked to relate every question to the study period only.

The first section contained seven closed yes-or-no questions about performed SARS-CoV-2 swabs, current or previous COVID-19, COVID-19 of household members, contact to SARS-CoV-2 infected or COVID-19 persons, current or previous quarantine and potential need of medical inpatient or intensive care treatment due to COVID-19.

The second section contained 14 questions about ophthalmological care and transport to their ophthalmologist during the study period: number of visits at their eye doctor’s office, eye hospital visits, performed intravitreal injections and the number of cancellations made by either the patients themselves or by their ophthalmologist.

In addition, patients could report the kind of transportation and any occurring problems.

The last section queried in seven closed yes-or-no questions if patients were:

- Living alone
- If and in case they did on which level they had official home-care support sponsored by the German health insurances (level 1 being the lowest with minimal need for assistance and level 5 being the highest with the lack of independence and extensive need for nursery service)
- If they had encountered COVID-19-related problems with mobile nursing service
- If they had help from relatives or friends in everyday care
- If government restrictions affected supply of daily needs.

### Statistics

Continuous variables were analysed with quartiles, as well as means and standard deviations. Categorical data were summarised with frequencies and proportions. Patient-related influences on cancelled dates were examined using one-dimensional logistic regression models. Data related to demography, care level, mobility and IVI were associated to the response variable of the number of refused and performed appointments. Odds ratios (and 95% confidence intervals) and *p* values were calculated. The probability of appointment cancellation was displayed to the range of values of the patients’ characteristics. In case of continuous data, the probabilities were displayed as line plots (ribbons for the 95% confidence interval), in case of categorical data as points (error bars for the 95% confidence interval). *p* values were two-sided, and the significance level was set to 5%. Data preparation, analyses and figures were created with R (R Core Team. 2020. R: A Language and Environment for Statistical Computing. Vienna, Austria: R Foundation for Statistical Computing).

## Results

### Patients

We included 1038 patients from Asklepios Nord-Heidberg (*n* = 307, 30%), Asklepios Barmbek (*n* = 397, 38%), Asklepios Altona (*n* = 126, 12%) and from the Department of Ophthalmology of the University Medical Center Hamburg-Eppendorf (UKE) (*n* = 208, 20%).

Twenty-four patients withdrew their consent or declined the questionnaire (*n* = 17) or else died after recruitment during our study period (*n* = 7) (none died of COVID-19) and were therefore excluded.

Eight hundred eighteen (78%) of the initial 1038 patients fully completed the questionnaire and were eligible for analysis.

Four hundred forty-two (53%) of these questionnaires were acquired by telephone, 307 (36%) in person (both in cooperation with a research assistant) and 93 (11%) by mail.

The proportional composition is as follows: AKN (*n* = 278; 34%), AKB (*n* = 366; 45%), AKA (*n* = 104; 13%) and UKE (*n* = 70; 8%) as shown in Table 1.

**Table 1:** Age, sex, duration of intravitreal injection (IVI) treatment and distribution of patients among the four hospitals of the recruited patients and number and proportion of questionnaires completely filled out and evaluated

	Recruited patients (*n* = 1038)	Completely answered questionnaire (*n* = 818)
Male	44% (*n* = 459)	44% (*n* = 359)
Age (years)	77 (± 10)	79 (± 10)
IVI treatment period (years)	3.1 (± 3,0)	3.1 (± 3,0)
AKN	30% (*n* = 307)	34% (*n* = 278)
AKB	38% (*n* = 397)	45% (*n* = 366)
AKA	12% (*n* = 126)	13% (*n* = 104)
UKE	20% (*n* = 208)	9% (*n* = 70)

Four hundred fifty-nine patients (56%) were female, 359 (44%) were male, and the mean age was 77 ± 10 years. In total, 1231 eyes (622 right eyes, 609 left eyes) were treated. Eight hundred twenty-eight (67%) had injections because of AMD, 189 (15%) because of DME, 179 (15%) because of macula oedema due to RVO and 35 (3%) because of other choroidal neovascularisations (CNV) (Table 2). Two thousand four hundred seventy-two intravitreal injections (1267 right eye, 1205 left eye) were performed during the period of March 15th and July 31st, 2020, in the study group. One hundred thirty-six (16%) of the patients received one, 216 (25%) two, 266 (31%) three, 184 (20%) four, 56 (6%) five and 15 (2%) six IVI during our study period (see Table 3).

**Table 2:** Diagnosis and number of treated eyes with intravitreal injection among the study period

Diagnosis	Treated eyes (*n* = 1231)
Neovascular AMD	67% (*n* = 828)
Diabetic macular oedema	15% (*n* = 189)
Retinal vein occlusion	15% (*n* = 179)
Other CNV	3% (*n* = 35)

**Table 3:** Number of intravitreal injections during the study period per patient

IVI during study period	Performed intravitreal injections (*n* = 2472)
1 IVI	16% (*n* = 136; OD 70, OS 66)
2 IVI	25% (*n* = 216; OD 113, OS 103)
3 IVI	31% (*n* = 266; OD 136, OS 130)
4 IVI	20% (*n* = 184; OD 85, OS 99)
5 IVI	6% (*n* = 56; OD 35, OS 21)
6 IVI	2% (*n* = 15; OD 8, OS 7)

The mean previous treatment period of the patients’ eye disease was 3.1 years ± 3.0, and the mean number of IVI received before the study period was 1.0 ± 11.0.

Ninety-seven percent of IVI were performed with anti-VEGF antibodies (33% aflibercept, 24% ranibizumab, 42% bevacizumab and 1% brolucizumab), 3% with dexamethasone (Ozurdex®).

### Questionnaire

One hundred one patients (12%) underwent COVID-19 swabs, of which four patients (0.5%) were tested positive. Two positive COVID-19 cases of people in the same household were found. Seven patients (1%) reported positive COVID-19 cases of a person they had been in closer contact, and 13 (2%) underwent quarantine. No patient required inpatient hospitalisation or intensive care due to COVID-19 infection. On average, patients had 3.9 ± 3.3 visits at their office-based ophthalmologist and 4.0 ± 3.0 visits at their treating eye hospital.

Seven hundred forty-eight patients (93%) cancelled no appointment, 37 (5%) one, 12 (1%) two and 10 (1%) cancelled three or more (up to 11), appointments at their office-based ophthalmologist themselves.

Thirty-four patients (7%) cancelled one, two or three appointments at their eye hospital.

Only 21 patients reported appointments cancelled by either their office-based ophthalmologist or by their hospitals. Thirty-two patients (4%) stated that one, 6 (1%) two, 1 (0,1%) three and 1 (0,1%) four IVI appointments were cancelled by themselves. Eight (1%) indicated that IVI appointments were cancelled by the eye hospitals.

Sixty-seven patients (8%) experienced longer ophthalmologist visits than before COVID-19 and 247 (31%) experienced shorter visits. Seven hundred ninety-six (99%) wore a protective face mask, and 59 (7%) additionally wore gloves during their ophthalmologist appointments.

To get to their appointments, 319 patients (39%) used public transportation, 167 (21%) used a taxi, 376 (47%) received help from relatives and 48 (6%) from travelling acquaintances during the journey (multiple answers possible). Problems of any kind with transportation were reported by 15 patients (2%). Three hundred eight patients (38%) reported to live alone, 363 (45%) were assisted in daily living by relatives, and 122 (15%) had limited mobility requiring a rollator or a wheelchair. One hundred four patients (13%) had an official care level: 29 (4%) care level 1, 51 (7%) care level 2, 21 (3%) care level 3, 2 (0.3%) care level 4 and 1 (0.1%) care level 5.

Eight patients (1%) experienced problems with outpatient nursing service due to COVID-19.

Cancellation rates to treatments by patients almost doubled in comparison to 2019 in the analysed time frame (94% increase).

### Correlations

Extensive correlation analyses were carried out over the entire data sets collected (Table 4).

**Table 4:** Probability of cancellation

Probability of cancellation:		
	Odds ratio (95% confidence interval)	*p* value
IVI treatment period	1.15 (1.09; 1.20)	*p* < 0.001
Age of patient	0.97 (0.96; 0.99)	*p* < 0.001
Low baseline visual acuity	1.26 (1.05; 1.51)	*p* < 0.001
COVID-19 infection in family environment	5.52 (2.68; 10.40)	*p* < 0.001
Increasing visual acuity of the non-treated eye	0.86 (0.55; 1.30)	*p* < 0.001
nAMD baseline diagnosis	0.64 (0.44; 0.92)	*p* = 0.016
DME baseline diagnosis	1.63 (1.02; 2.52)	*p* = 0.033
RVO baseline diagnosis	1.10 (0.67; 1.73)	*p* = 0.688
Other baseline diagnoses	1.72 (0.66; 3.69)	*p* = 0.208
Bilateral IVI during study period	0.87 (0.56; 1.41)	*p* = 0.541
Immobility	0.43 (0.20; 0.81)	*p* = 0.016
Living alone	0.69 (0.46; 1.01)	*p* = 0.065
Care level	0.59 (0.26; 1.14)	*p* = 0.148
Use of public transport and taxi	0.89 (0.62; 1.28)	*p* = 0.538
Support of relatives	1.00 (0.69; 1.44)	*p* = 0.994
Report of home supply problems	0.42 (0.02; 1.92)	*p* = 0.391
Gender	1.09 (0.76; 1.60)	*p* = 0.630

**Fig. 1:**
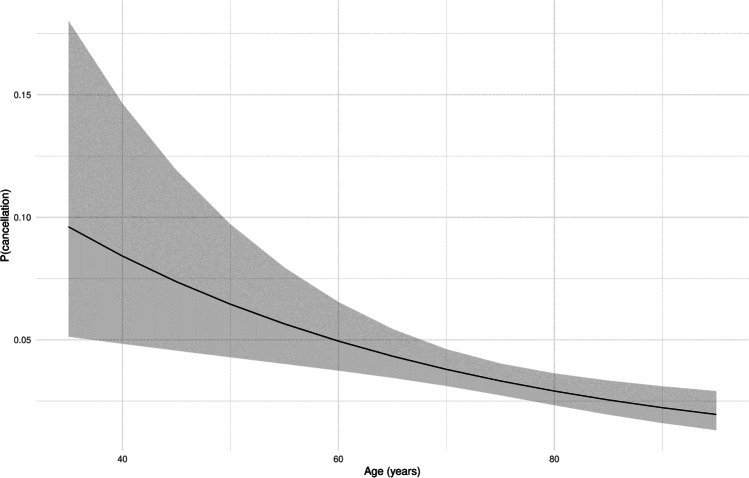
Probability of cancellation correlated to IVI treatment period (years); plot effect (black line, calculated probability; grey ribbon, 95% confidence interval)

**Fig. 2:**
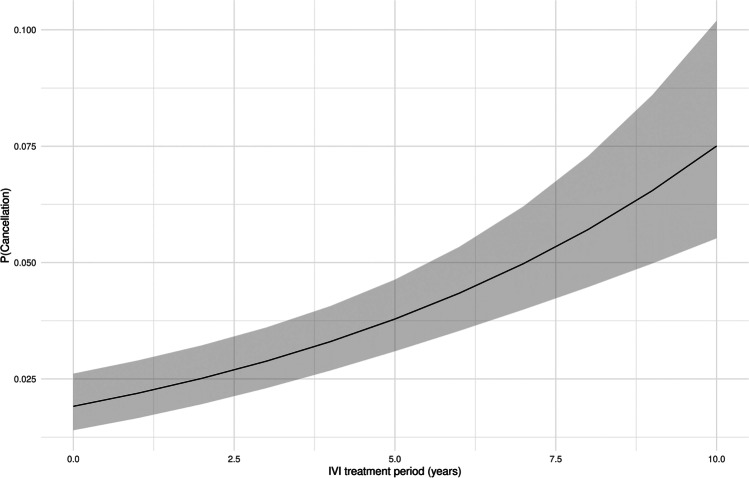
Probability of cancellation correlated to age (years); plot effect (black line, calculated probability; grey ribbon, 95% confidence interval)

Longer duration of intravitreal therapy before the study period was associated with a higher probability of visit cancellation. Each additional year of intravitreal treatment increased the odds of cancellation (Fig. 1) (odds ratio (OR) was 1.15 [1.09; 1.20] (*p* < 0.001)). On the other hand, every additional year of life posed a 2.6% lower risk of visit cancellation (Fig. 2), so older patients showed better compliance (OR 0.97 [0.96; 0.99] (*p* < 0.001)).

A COVID-19 infection in the family environment displayed a 5.5-fold chance of visit cancellation (estimate, confidence interval 2.68–10.42). The OR of visit cancellation in a COVID-19-related environment compared to a no COVID-19-related environment was 5.52 [2.68; 10.40] (*p* < 0.001).

**Fig. 3:**
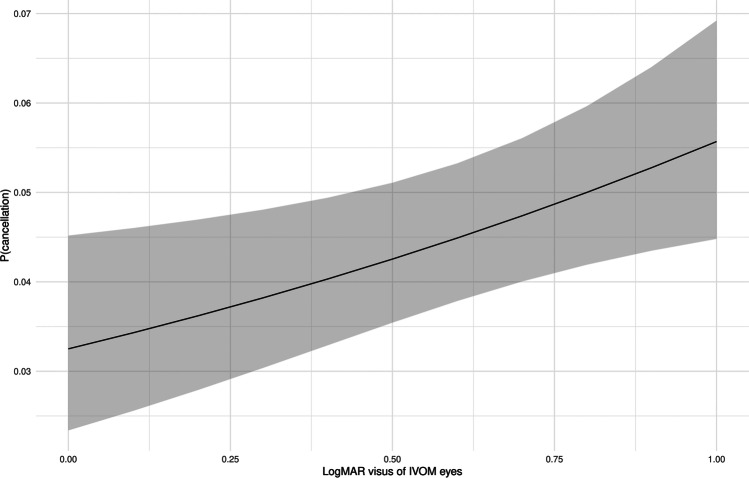
Probability of cancellation correlated to visual acuity (logMAR BCVA) of the treated eye; plot effect (black line, calculated probability; grey ribbon, 95% confidence interval)

Lower baseline visual acuity of the treated eye was significantly associated with a higher chance of cancellation, as shown in Fig. 3 (OR 1.263 [1.05; 1.51] (*p* < 0.001).

**Fig. 4:**
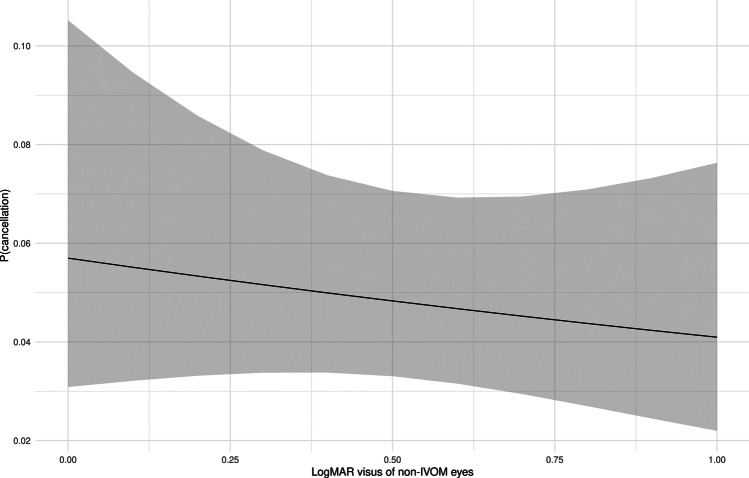
Probability of cancellation correlated to visual acuity (logMAR BCVA) of the fellow untreated eye; plot effect (black line, calculated probability; grey ribbon, 95% confidence interval)

The cancellation rate decreased with an decreasing BCVA of the non-treated eye, as seen in Fig. 4 (OR 0.866 [0.55; 1.30] (*p* < 0.001).

Immobility and living alone showed a reduced risk of visit cancellation (OR 0.43 [0.20; 0.81] (*p* = 0.016) and 0.69 [0.46; 1.01] (*p* = 0.065), respectively). An existing care level did not reduce the risk of visit cancellation with statistical significance (OR 0.59 [0.26; 1.14] (*p* = 0.148).

The use of public transport and taxis (OR 0.893 [0.621; 1.28] (*p* = 0.538)) as well as support of relatives (OR 1.00 [0.69; 1.44] (*p* = 0.994)) did not influence visit cancellation significantly and neither did the report of home supply problems (OR 0.42 [0.02; 1.92] (*p* = 0.391)).

Comparing baseline diagnosis, patients with nAMD had a 36% reduced risk of visit cancellation (OR 0.64 [0.44; 0.92] (*p* = 0.016). Patients treated because of DME cancelled their appointments more often (OR 1.63 [1.02; 2.52] (*p* = 0.033).

The diagnosis of retinal vein occlusion (RVO) (OR 1.10 [0.67; 1.73] (*p* = 0.688)) and other choroidal neovascularization (CNV) (OR 1.72 [0.66; 3.69] (*p* = 0.208)) did not reach statistical significance. Bilateral IVI or gender also showed no statistical significance regarding visit cancellation (OR 0.87 [0.56; 1.41] (*p* = 0.541) and OR 1.09 [0.76; 1.60] (*p* = 0.630)), respectively.

Due to the small number of manifest COVID-19 (*n* = 4) among our cohort, no statistical impact could be detected.

## Discussion

This study identified multiple factors significantly affecting the probability of visit cancellation during the COVID-19 pandemic.

A long preceding treatment period, a COVID-19 infection in the family environment and DME could be identified as risk factors for IVI cancellations and consequently reduced medical care.

The fact that when treating nAMD an initially achieved increase in visual acuity during the first couple of years cannot be maintained in the long term may have resulted in higher cancellations among patients with a longer treatment period [21, 22].

A COVID-19 infection in the immediate environment resulted in higher cancellations. This might not come as a surprise and is probably caused by the fear of either spreading or acquiring the disease or the possible need to quarantine.

During the study period, we observed a decrease in compliance among patients with lower visual acuity at baseline. This has also been described before COVID-19 [23]. It can be assumed that this is the case because visual impairment is a disability that creates constraints in various aspects of daily life such as self-care and accessing medical care [24] or patients might have been already more frustrated because of their vision and disease status.

Additionally, we found that a better visual acuity of the fellow untreated eye also resulted in reduced compliance.

We did not specifically ask, but it can be speculated that there was a reduced sense of urgency for treatment in the patients with good vision of the fellow eye, while anxiety about acquiring the virus increased.

Decreased compliance among diabetic patients being treated with IVI because of DME is well-known and was described before the pandemic as well [25, 26]. Ehlken et al. have already shown that DME patients have a significantly higher nonadherence already over a treatment period of 1 year than, e.g. nAMD patients (44% vs 32%) [27]. The higher comorbidities in diabetes patients are assumed to be one major reason for this, but a detailed analysis on this difference is still lacking. This was consistent during our study which showed that patients treated because of DME cancelled their appointments significantly more often. This also matches the findings of Borelli et al. [19]. In comparison, nAMD showed a 36% reduced risk of cancellations compared to DME, RVO and other CNV.

Interestingly and unexpectedly, higher age showed to have a protective effect regarding the risk of cancellation of intravitreal injections. This is in contrast to the assumption that older age per se leads to poorer care because of comorbidities and higher vulnerability.

It also shows that age cannot be equated with IVI treatment duration when looking at medical care. Immobility, a known risk factor for poorer medical care surprisingly, led to less cancellation during COVID-19 pandemic. This might be due to the fact that the pandemic was a rapidly accepted crisis with broad fear for decreased medical care and that therefore acquaintances and healthcare workers paid more attention to the more vulnerable members of society.

Heimes et al. investigated the influence of telemedicine approaches in the IVI patients. Patients were divided into two groups: one group was monitored close to home vs. the second group was monitored in tertiary centres. It was shown that the group with follow-up close to home showed better adherence than the patients examined directly in tertiary centres [28]. This method represents a possibility for more rural areas to improve patient adherence in the future, but in our environment of a metropolitan region with approximately 150 ophthalmologists in the area of the city of Hamburg, we do not expect a greater effect through telemedical projects with regard to IVI controls. Further developments in this area remain to be seen, and the possibility of providing OCT examinations with a home office device in the future could improve the adherence and treatment quality. The ease of use for our old and visually impaired patients would be a top priority for such devices.

Other than assumed and previously described by Droege et al., transportation did not impact cancellation [29] in our data set.

Furthermore, living alone, an apparent level of care and home supply problems did not influence the rate of cancellation significantly.

Bilateral, not simultaneously, administered IVI during our study period also showed no impact on cancellation. Data collected so far on the effect of bilateral intravitreal injection therapy is conflicting. Studies worked out bilateral therapy to result in increased and decreased compliance [30].

According to our knowledge, this study analysed the biggest number of eyes (1231) and patients (1038) during COVID-19 regarding IVI compliance. Wasser et al., having completed the largest study so far, included 636 eyes *during* a 1-month study period [18].

Because of the 20-week study period, no distinction was made between the terms nonadherence and nonpersistence; a homogeneous definition in literature is also missing [28].

Limiting factors of this study were that no data of the course and potential gain or loss of visual acuity was collected as well as the fact that the BCVA was measured in Snellen equivalent and not in a standardised study settings (e.g. ETDRS). This study mainly analysed the parameters of the total population of IVI patients. The distribution of indications is similar to that found in population-based studies with 2/3 AMD patients and 1/3 for the remaining indications. The analyses and statements we have made here are therefore mainly statements for the entire group; a highly specific subgroup analysis was not carried out due to the small number of patients in the non-AMD group.

## Conclusion

Our study shows that the eye hospitals in the city area of Hamburg were able to maintain and continue IVI therapy despite the tightened situation with restricted capacities and under difficult general conditions. Nevertheless, cancellation of visits by the patients was a major issue.

IVI are highly important because of the potential risk of vision loss and reduced quality of life. From a patient’s point of view, during the SARS-CoV-2 pandemic, IVI might not have had the same relevance and urgency for treatment as life-threatening diseases. It is therefore mandatory to inform each patient about the necessity of regular treatment even under the conditions of a pandemic.

With the newly described risk factors, patients at risk can be detected and proper arrangements made for a more personalised and improved individual medical care.

One real-life application could be to call after a patient’s treatment with IVI due to nAMD for more than 2 years as well as the use of longer lasting intravitreal corticosteroids when treating DME during times of reduced medical care.

This study was started during a period of uncertainty about the course of the pandemic, the influence of both medical and governmental measures and most important before the implementation of SARS-CoV-2 vaccination. It will be interesting to analyse its effect on intravitreal injections and medical care in the future and also its prospective long-term consequences.

## Supplementary Information

Below is the link to the electronic supplementary material.

Supplementary file1 (DOCX 31 KB)

## Abbreviations

AKA: Asklepios Hospital Altona
AKB: Asklepios Hospital Barmbek
AKH: Asklepios Hospital Nord-Heidberg
AMD: Age-related macular degeneration
BCVA: Best-corrected visual acuity
CNV: Choroidal neovascularisation
DME: Diabetic macular oedema
IVI: Intravitreal injection
P (cancellation): Probability of cancellation
RVO: Retinal vein occlusion
UKE: University Medical Center Hamburg-Eppendorf
