# Supplementary material for: Impact of the COVID-19 pandemic’s first wave on the care and treatment situation of intravitreal injections in a German metropolitan region
Source: Graefes Arch Clin Exp Ophthalmol. 2022 Jan 10;260(6):1877–86. doi: 10.1007/s00417-021-05521-5 (PMC8743739; doi:10.1007/s00417-021-05521-5)
Supplement: Supplementary file 1 — Supplementary file1 (DOCX 31 KB) [file 417_2021_5521_MOESM1_ESM.docx]

**
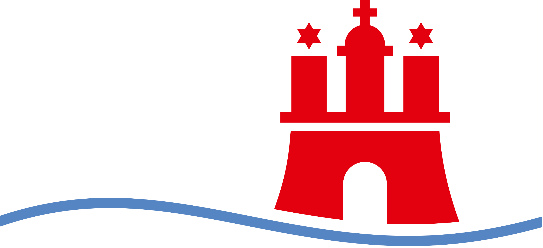
Care and treatment situation of patients receiving intravitreal injections in the city of Hamburg**

**Questonnaire**

| **age** |  | years |
| --- | --- | --- |
| **sex** | male | female |
| **start of intravitreal therapy** | year: |  |
| **date (filling in of questonnaire)** | date: |  |

**Please fill in all questions in this questonnaire as precisely as possible.**

**By filling in the questonnaire you help us receiving new data on patient care and treatment in this special situation.**

**Thank you for your participation!**

| **Questions concerning COVID-19:** |  |  |  |  |
| --- | --- | --- | --- | --- |
| Was a COVID-19 nasal swab performed? | yes | no | date: |  |
| Have you been infected with SARS-CoV-2 during the study period? | yes | no | date: |  |
| Was a relative/household member in your family environment infected with SARS-CoV-2? | yes | no | date: |  |
| Was a contact person (contact more than 15minutes, distance 2m or less) tested positive for SARS-CoV-2? | yes | no | date: |  |
| Have you been in quarantine? | yes | no | date: |  |
| Have you been hospitalised because of a SARS-CoV-2 infection? | yes | no | date: |  |
| If yes: Did you need intesive care or mechanical ventilation? | yes | no | date: |  |

| **Questions concering ophthalmological care in study period:** | | |
| --- | --- | --- |
| How many times did you see your eye doctor (office)? | number: |  |
| How many times did you visit your eye hospital? | number: |  |
| How many appointments at your eye doctors`s office did you cancel? | number: |  |
| How many appointments at your eye hospital did you cancel? | number: |  |
| How many appointments for intravitreal injections did you cancel? | number: |  |
| How many appointments for intravitreal injections were cancelled by your eye hospital? | number: |  |
| How many ophthalmological control visits have been cancelled due to COVID-19? | number: |  |
| How many intravitreal injections did not take place due to COVID-19? | number: |  |
| Were visits at your eye doctors`s more time consuming than normal? | yes | no |
| Were visits at your eye doctors`s less time consuming than normal? | yes | no |
| Did your were a mouth nose mask during your visits? | yes | no |
| Did your were protective gloves during your visits? | yes | no |
| How did you get to your eye doctor/your eye hospital? (multiple answers possible) | Public transport | taxi |
|  | relatives | friends |
| Did you experience problems during your journey to your eye doctor/your eye hospital? | yes | no |

**
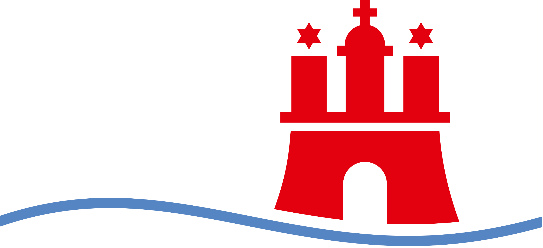
**

| **Questions concerning health and care situation:** | | |
| --- | --- | --- |
| Do you live alone? | yes | no |
| Do you need to use a wheelchair or a rollator during your visits? | yes | no |
| Do you have a care level? | yes | no |
| If yes: which care level do you have? | care level: |  |
| If you rely on nursing services: Did any problems occur during the study period? | yes | no |
| Did relatives or friends support you during the study period additionally? | yes | no |
| Did you experience problems in the supply of daily needs because of closed shops? | yes | no |
